# Neuromyelitis Optica Diagnosis in Two Elderly Patients with Systematic Lupus Erythematosus: A Case Series

**DOI:** 10.3390/reports8030110

**Published:** 2025-07-16

**Authors:** Kyriaki Astara, Maria Lypiridou, Konstantinos Kalafatakis, Georgios Nikolaou, Georgios Stouraitis

**Affiliations:** 1 Department of Neurology, Army Share Fund Nursing Institution (NIMTS), 11521 Athens, Greece; marialypiridou@gmail.com (M.L.);; 2 Department of Neurology, General Hospital of Athens “Pammakaristos”, 11144 Athens, Greece; k.kalafatakis@uoi.gr; 3 Human Computer Interaction Laboratory, University of Ioannina, 47150 Arta, Greece

**Keywords:** neuromyelitis optica, immunology, case report

## Abstract

**Background and Clinical Significance:** Neuromyelitis optica (NMO) is a chronic demyelinating inflammatory disease of the central nervous system (CNS), mediated by autoantibodies against aquaporin-4 (AQ4) receptors. In the spectrum of NMO, other autoimmune diseases also coexist, though their association with systemic lupus erythematosus (SLE) is rare. **Case Presentation:** We present two cases of patients in their 70s who were diagnosed with NMO in the context of SLE. The first case concerns a 78-year-old woman with drug-induced SLE and thoracic myelitis who developed T4-level incomplete paraplegia over three weeks. The second case involves a 71-year-old woman with a history of SLE and myasthenia gravis, presenting with cervical myelitis with progressive worsening of walking and C4-level paraparesis over two months. In both cases, elevated serum anti-AQ4 titers were detected, establishing the diagnosis of NMO and differentiation from an atypical manifestation of SLE-related myelitis. High doses of intravenous corticosteroids with gradual tapering, along with cyclophosphamide, followed by rituximab, were administered in both patients. The first patient showed a poor response, while the second showed improvement. **Conclusions:** The coexistence of NMO with SLE is rare, but the occurrence of myelitis in patients with connective tissue diseases should raise the suspicion of NMO, especially in elderly women and several years after the diagnosis of SLE. Time to treatment is critical, as delays in treating NMO can result in cumulative and disabling damage.

## 1. Introduction and Clinical Significance

Neuromyelitis optica (NMO) is a central nervous system (CNS) autoimmune disorder characterized by optic neuritis and longitudinally extensive transverse myelitis, typically involving more than three contiguous spinal cord segments. The disease is associated with pathogenic IgG antibodies targeting aquaporin-4 (AQ4) water channels on astrocyte podocytes [1]. Systematic lupus erythematosus (SLE), a multisystem autoimmune disorder, may involve the CNS, occasionally presenting with myelitis. However, myelitis in SLE affects fewer vertebral segments, has a monophasic course, and tends to present earlier in life than NMO [2].

We report two rare and diagnostically complex presentations of neuromyelitis optica spectrum disorder (NMOSD) in two elderly female patients with SLE, illustrating the rare coexistence of these conditions and highlighting diagnostic complexities involved.

## 2. Case Presentation

***Case 1:*** A 78-year-old Caucasian female with a medical history of type 2 diabetes mellitus presented with a sudden-onset three-week history of progressive gait. Neurological examination revealed preserved higher cortical functions and cranial nerves II–XII but demonstrated quadriparesis, with proximal lower limb weakness (2/5), distal lower limb weakness (4/5), and upper limb weakness (3/5), along with hyperreflexia in the lower limbs and bilateral Babinski sign. Tone was normal, but muscle atrophy was present in both the trapezoids and vastus muscles of the left foot. Abdominal reflexes were absent. A level of sensory perception change at T6 was indicated, and urinary retention was reported. Her modified Rankin Scale (mRS) score at admission was 5, while before the onset of symptoms, it was 0. Initial laboratory and imaging (CT brain and lumbar spine) were unremarkable. Laboratory findings were significant for normocytic anemia. The patient was admitted to the Neurology Department for further investigation.

**Figure 1:**
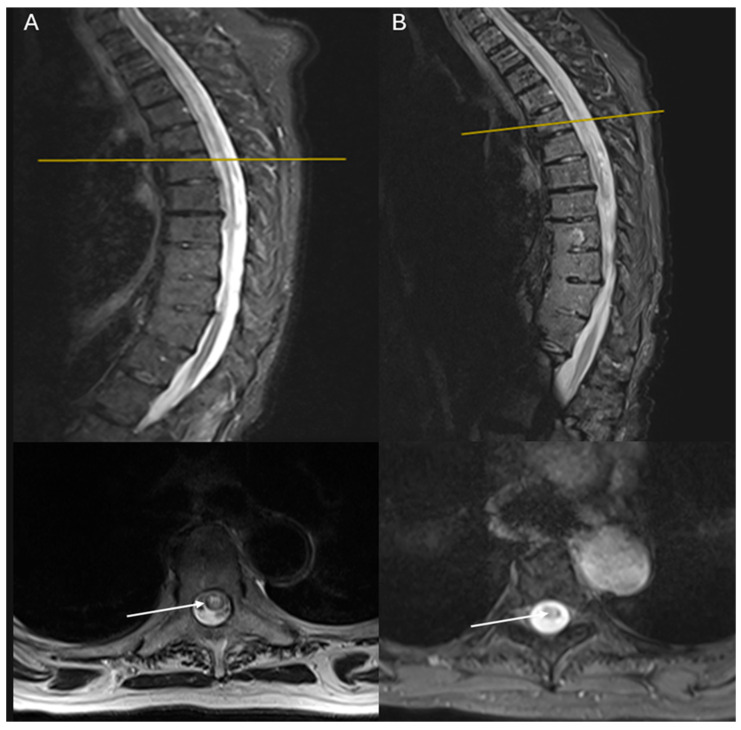
MRI imaging of the first case demonstrating a long segment of a well-defined lesion extending from T3 to T8, appearing low on T1 with no gadolinium enhancement and high on T2:(**A**) sagittal and axial T2 STIR-weighted MRI images of the thoracic spine at admission; (**B**) sagittal and axial T2 STIR-weighted MRI images of the thoracic spine 1 year of follow-up. White arrows show the lesions of hyperintensities involving the central and the right half of the cord, sparing the dorsolateral part. One year later, lesions had the same extent but weaker T2 hyperintensity.

She underwent an electrodiagnostic investigation, which showed mild left carpal tunnel syndrome and non-specific low muscle contraction of the lower extremities. CT scans of the cervical and thoracic spine were also performed, with unremarkable results. Magnetic resonance imaging (MRI) scans of the brain and cervical spine showed no significant results, while the thoracic spine demonstrated a well-defined, multi-segment lesion extending from T3 to T8, having low-intensity signal on T1-weighted imaging with no gadolinium enhancement and high-intensity signal on T2-weighted imaging (Figure 1). Lumbar puncture (LP) showed no abnormal results (Table 1). Serum anti-AQ4 antibodies were positive, using an enzyme-linked immunosorbent assay (ELISA) (320 U/mL). Extended autoimmune workup showed elevated anti-nuclear antibodies (ANAs) (157.00 AU/mL) and ds-DNA antibody (97.90 IU/mL), raising suspicion for lupus spectrum disease. Normocytic anemia was attributed to autoimmune hemolysis, as indicated by elevated LDH and reticulocytes, along with a positive direct Coombs test. Given the patient’s history of prior antibiotic exposure, drug-induced lupus was initially considered; however, the presence of high-titer ANA, ds-DNA positivity, and the absence of anti-histone antibodies, along with the involvement of the CNS, pointed to SLE. The diagnosis was supported by the EULAR 2019 Classification Criteria for SLE, which require positive ANA as an entry criterion, followed by the involvement of the hematologic domain, due to autoimmune hemolysis [3]. Cerebrospinal fluid (CSF) PCR was positive for HHV-6 infection; the patient received intravenous acyclovir accordingly. Cancer biomarkers and thyrotropin were normal. Hence, a diagnosis of NMO with SLE was documented.

The patient was initially treated with intravenous infusions of 1gr methylprednisolone, which was discontinued due to a pancreatic reaction, followed by two cycles of 1gr of cyclophosphamide. Then, she was scheduled for semiannual sessions of intravenous infusion of 1gr RTX.

One year later, the patient remained functionally dependent (mRS 5), with stable MRI findings and decreased anti-AQ4 titers (100 U/mL) (Table 1).

***Case 2:*** A 71-year-old Caucasian female with a medical history of arterial hypertension, prior thyroidectomy (on levothyroxine) and a history of SLE (with musculoskeletal and hematologic involvement of joint pain and morning stiffness, and autoimmune hemolytic anemia-treated with hydroxychloroquine and methylprednisolone) presented with a 2-month history of sudden-onset progressive gait disturbance, neuropathic pain in the lower extremities, and urinary incontinence. Neurological examination revealed preserved cognitive function and cranial nerves II–XII, with paraparesis more pronounced on the left (4/5 proximally, 3/5 distally with drop foot) than on the right (4/5) and normal muscle strength of upper extremities. Reflexes were brisk in the left lower limb, while bilateral Hoffman and Babinski signs were present. Tone was normal in all extremities. Sensory findings included reduced pinprick sensation below C3 and impaired proprioception in the right lower limb. Her mRS at presentation was 4, while before the onset of symptoms, it was 0. The laboratory evaluation of biofluids on admission was unremarkable. She was admitted to the Neurology Department for further investigation.

**Figure 2:**
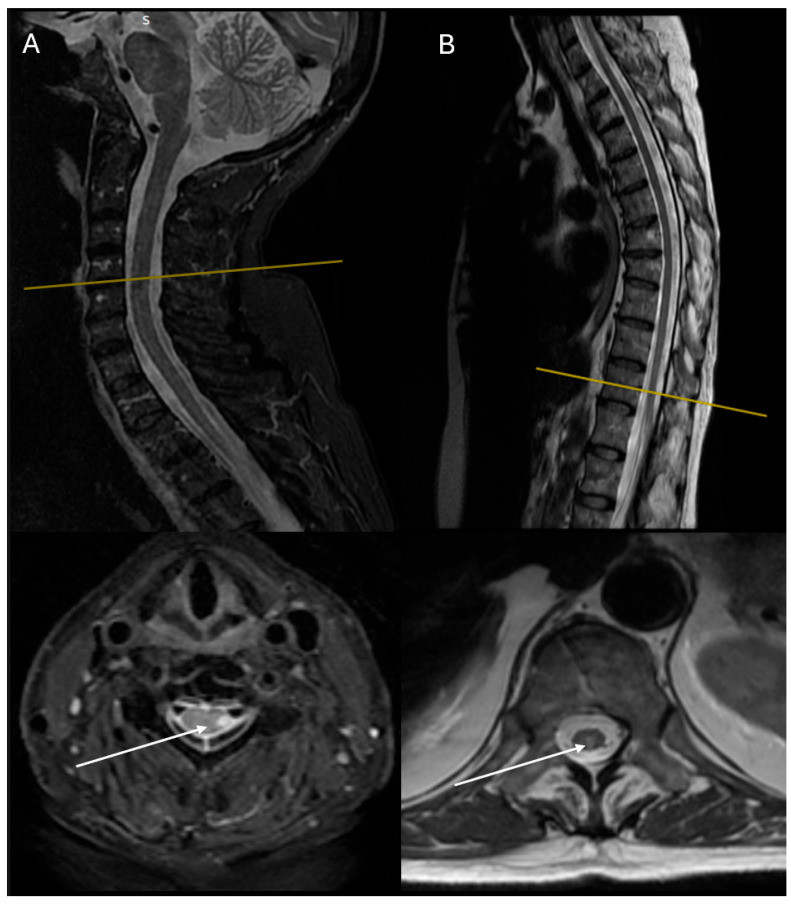
MRI imaging of the second case demonstrating a long segment of a well-defined lesion extending from C3 to C7, appearing low on T1 with no gadolinium enhancement and high on T2: (**A**) sagittal and axial T2 STIR-weighted MRI images of the cervical spine at admission; (**B**) sagittal and axial T2-weighted MRI images of the thoracic spine 1 year of follow-up. White arrows show the lesions of hyperintensities involving the dorsolateral part of the cord. One year later, lesions were increased in number but reduced in extent, while T2 hyperintensities were weaker as well.

Outpatient MRI scans of the cervical and lumbar spine demonstrated a well-defined, multi-segment lesion extending from C3 to C7, having low-intensity signal on T1-weighted imaging with no gadolinium enhancement and high-intensity signal on T2-weighted imaging (Figure 2). She underwent additional MRI scans of the brain and thoracic spine (no notable findings), while LP showed no abnormal findings (Table 1). Serum anti-AQ4 receptor antibodies were markedly elevated, using ELISA (3200 U/mL), while extended serological workup was only indicative of SLE. Hence, a diagnosis of NMO with SLE was documented.

The patient was treated with intravenous infusions of 1gr methylprednisolone for 5 days, followed by two cycles of 1gr cyclophosphamide while continuing with tapering oral steroids. Then, she was scheduled for semiannual sessions of intravenous infusion of 1gr RTX.

One year later, the patient showed significant clinical improvement (mRS 1), with minimal residual left lower limb weakness (4/5). Follow-up cervical and thoracic spine MRI displayed reduced lesion extent with a weaker increase in T2 signal intensity and no gadolinium enhancement (C4–C7), though a new demyelinating lesion extending to T9–T12 was detected. Anti-AQ4 receptor antibody titer declined to 100 U/mL (Table 1).

## 3. Discussion

In this paper, we present two cases of SLE and NMO, highlighting the heterogeneity in disease onset, progression, and response to treatment. The first case was initially suspected to be drug-induced lupus, due to prior antibiotic use [4]. Nevertheless, persistent neurological symptoms months after discontinuation of the offending agents, relevant coexistent clinical domains, and the serological profile suggested an alternative diagnosis [5]. The second case, with a prior diagnosis of SLE, was maintained on hydroxychloroquine and low-dose methylprednisolone. The first case presented with a rather abrupt and severe NMO onset, with limited therapeutic response, whereas the second exhibited gradual progression and excellent response to the same standard immunosuppressive therapy. One could attribute the gradual progression and favorable response of the second case to the immunosuppressive effect maintained methylprednisolone. Several factors have been associated with poor prognosis, including age >50 years, female sex, African ethnicity, late pregnancy/postpartum period, high AQ-4 antibody titers, longitudinally extensive transverse myelitis, and spinal cord atrophy [6,7].

A recent systematic review of 46 cases with NMOSD in SLE demonstrated female predominance and NMO onset after lupus, as in our cases, but earlier age of onset and at least one relapse after treatment [8]. To our knowledge, these are the first reported cases of SLE with late-onset NMO and such a different clinical course.

Immunosuppressive therapy is fundamental for NMO management. In particular, RTX, a CD20-targeting monoclonal antibody, depletes CD20+ B cells, suppressing antibody-mediated immunity and reducing AQ4 antibodies, which have been implicated in the pathogenesis of NMO [9]. In fact, AQ4 antibodies demonstrate 94% specificity and 76% sensitivity for NMOSD [10]. RTX is a highly efficient option in reducing relapse rates and long-term disability, while it is well tolerated by patients, despite the lack of large randomized controlled trials [11,12].

A subset of patients, such as our first case, have shown a limited response to RTX. In a study by Dai et al. [13], NMO cases with suboptimal response to RTX were reviewed, proposing possible mechanisms like suboptimal depletion, the production of neutralizing antibodies of several B cell subsets [14], the involvement of cell-mediated immunity by various T cell subsets [15], and complement activation [16]. In such cases, tocilizumab (anti-interleukin-6 receptor) and eculizumab (complement inhibitor) have displayed promising results [16,17]. However, to our knowledge, refractory NMO in the context of SLE has not been previously reported.

## 4. Conclusions

Although rare, the coexistence of SLE with NMO should be considered in patients with connective tissue disorder presenting with myelitis, especially in women over 65 years of age, even several years after the diagnosis of SLE. Prompt recognition is crucial, as time-to-treatment is critical and may result in irreversible neurological damage. While RTX therapy is generally effective, clinical vigilance is essential for cases with suboptimal response.

## Figures and Tables

**Table 1:** Overview of the two cases. Abbreviations: NMOSD = Neuromyelitis optica spectrum disorder; LP = lumbar puncture; AQ4 = aquaporin-4; CYC = cyclophosphamide; RTX = rituximab; EDSS = Expanded Disability Status Scale.

	*Case 1*	*Case 1*—Follow-Up	*Case 2*	*Case 2*—Follow-Up
**Age of NMOSD Diagnosis**	78	79	71	72
**Level**	T3–T8	T3–T8	C3–C7	C4–C7, T10–T12
**Neurological Manifestation**	Incomplete paraparesis T4 level	Indifferent	Incomplete paraparesis C4 level, worse on the left side	Improved—slight residual weakness on the left lower extremity distally
**LP:**	0 cells, Glucose: 62 mg/dL, Protein: 69 mg/dL, LDH: 51, culture (−) cytology (−) for malignancy	-	0 cells, Glucose: 51 mg/dL, Protein: 32 mg/dL, LDH: 46, culture (−) cytology (−) for malignancy (presence of cellular elements compatible with the inflammatory process)	-
**Oligoclonal Bands**	Type 3	-	Type 3	-
**Anti-AQ4**	320	100	3200	100
**Significant Workup**	PCR HHV-6 (+) AΝA (+) and dsDNA (+)	-	↓C3: 71, ↓C4: 4	-
**Treatment**	Iv methyl-prednisolone 1gr × 5 days -> stop due to pancreatic reaction 2 cycles CYC	2 cycles RTX	Iv methyl-prednisolone 1gr × 5 days, with gradual per os tapering up to 16 mg. 2 cycles CYC	2 cycles RTX
**EDSS**	7.5	7.5	7	4
**Outcome**	Stagnant—bedridden		Improved—able to stand and walk with support and self-service capability	

## Data Availability

The original contributions presented in this study are included in the article. Further inquiries can be directed to the corresponding author.

## Abbreviations

The following abbreviations are used in this manuscript:

NMOSD	Neuromyelitis optica spectrum disorder
SLE	Systematic lupus erythematosus
AQ4	Aquaporin-4
mRS	Modified Rankin Scale
ECG	Electrocardiogram
CT	Computed tomography
MRI	Magnetic resonance imaging
CSF	Cerebrospinal fluid
LP	Lumbar puncture
ELISA	Enzyme-linked immunosorbent assay
CYC	Cyclophosphamide
RTX	Rituximab
EDSS	Expanded Disability Status Scale
